# A whey-protein supplement increases fat loss and spares lean muscle in obese subjects: a randomized human clinical study

**DOI:** 10.1186/1743-7075-5-8

**Published:** 2008-03-27

**Authors:** Joy L Frestedt, John L Zenk, Michael A Kuskowski, Loren S Ward, Eric D Bastian

**Affiliations:** 1 Minnesota Applied Research Center (MARC), Edina, MN, USA; 2 Geriatric Research Education and Clinical Center (GRECC), Veterans Administration Medical Center, Minneapolis, MN, USA; 3 Glanbia Research and Development Center, Twin Falls, ID, USA

## Abstract

**Background:**

This study evaluated a specialized whey fraction (Prolibra™, high in leucine, bioactive peptides and milk calcium) for use as a dietary supplement to enhance weight loss.

**Methods:**

This was a randomized, double-blind, parallel-arm, 12-week study. Caloric intake was reduced 500 calories per day. Subjects consumed Prolibra or an isocaloric ready-to-mix beverage 20 minutes before breakfast and 20 minutes before dinner. Body fat and lean muscle tissue were measured by dual-energy x-ray absorptiometry (DEXA). Body weight and anthropometric measurements were recorded every 4 weeks. Blood samples were taken at the beginning and end of the study. Statistical analyses were performed on all subjects that completed (completer analysis) and all subjects that lost at least 2.25 kg of body weight (responder analysis). Within group significance was determined at *P *< 0.05 using a two-tailed paired t-test and between group significance was determined using one way analysis of covariance with baseline data as a covariate.

**Results:**

Both groups lost a significant amount of weight and the Prolibra group tended to lose more weight than the control group; however the amount of weight loss was not significantly different between groups after 12 weeks. Prolibra subjects lost significantly more body fat compared to control subjects for both the completer (2.81 vs. 1.62 kg *P *= 0.03) and responder (3.63 vs. 2.11 kg, *P *= 0.01) groups. Prolibra subjects lost significantly less lean muscle mass in the responder group (1.07 vs. 2.41 kg, *P *= 0.02). The ratio of fat to lean loss (kg fat lost/kg lean lost) was much larger for Prolibra subjects for both completer (3.75 vs. 1.05) and responder (3.39 vs. 0.88) groups.

**Conclusion:**

Subjects in both the control and treatment group lost a significant amount of weight with a 500 calorie reduced diet. Subjects taking Prolibra lost significantly more body fat and showed a greater preservation of lean muscle compared to subjects consuming the control beverage. Because subjects taking Prolibra lost 6.1% of their body fat mass, and because a 5% reduction of body fat mass has been shown to reduce the risk of obesity related disease, the results have practical significance.

## Introduction

The growing obesity epidemic is a world wide concern [1]. Obesity contributes to health issues that result from carrying increased fat mass such as sleep apnea, osteoarthritis and joint and skin abnormalities and health issues that result from the metabolic effect of fat cells such as type 2 diabetes, insulin resistance, metabolic syndrome, hypertension, nonalcoholic fatty liver disease, heart disease, gallbladder disease and cancer [2,3]. Decreasing body fat mass in humans significantly reduces health issues that arise from increased body fat [2,3].

An effective approach to weight management is to increase dietary protein or change the ratio of carbohydrate to protein in the diet [4]. A low carbohydrate to protein ratio (<2) with greater than 100 grams of protein per day in the form of meats, eggs, cheese, milk and nuts increased fat loss and retained lean muscle during dieting [5-8]. Layman et al. [5] studied body composition and weight loss in women who consumed a hypocaloric diet with a 1.4 or a 3.5 carbohydrate to protein ratio. Weight loss was not significantly different between groups but fat loss significantly increased for those consuming the high protein diet (~125 g/day). Skov et al. [8] measured the effect of carbohydrate to protein ratio on body composition in a hypocaloric study. The treatment group consumed an *ad libitum *fat-reduced diet with a 1.9 carbohydrate to protein ratio and the control group consumed an *ad libitum *fat reduced diet with a 4.9 carbohydrate to protein ratio. The weight and fat losses over six months significantly increased in the high protein group compared to the control group (8.0 kg versus 5.1 kg for weight loss and 7.6 versus 4.3 kg for fat loss).

Increasing fat loss through dietary changes helps retain lean muscle mass. Retaining lean muscle translates into increased body strength, increased basal metabolic rate and increased bone strength [9]. The retention of lean muscle during weight loss may be related to the leucine's ability to stimulate muscle synthesis [10]. The post-prandial rate of protein synthesis also depends on the speed of protein absorption. Fast absorbing protein has an anabolic effect [11]. The high leucine content (50–75% more than other common food proteins) of whey proteins [10] coupled with fast absorption [11] make whey protein ideal as a protein supplement during weight loss.

Whey proteins also modulate several hormones that influence body composition. Short term acute studies with whey proteins corroborate the body composition changes seen with longer term feeding studies. Whey protein isolate (75 grams per dose) was evaluated [12] for its impact on obesity-related hormones in an acute (5 hour) protein ingestion in overweight and obese women with polycystic ovary syndrome (PCOS). The acute hormonal response showed significantly lower hyperinsulinemia (less lipogenesis), lower cortisol levels (lean muscle preservation) and increased ghrelin release (satiety enhancement).

Another dietary approach to decrease body fat is to increase dietary calcium. Increasing dietary calcium decreased body fat and improved body composition in several studies [13-20]. Two different mechanisms have been suggested and include the formation of calcium soaps and decreased intestinal absorption of fat [21,22] or an indirect hormonal mechanism [19] that increases lipolysis in adipocyte tissue.

Several studies [23,24] show that calcium supplementation with dairy products may arrest bone resorption during weight loss, provide stronger bones and reduce the potential for fractures after weight loss particularly in women over 65. Women over 65 who lose weight are at least 1.8 times more likely to have a bone fracture compared to counterparts that do not lose weight [25]. Other benefits of dairy minerals include research showing that milk minerals decrease co-morbidities that are associated with obesity such as hypertension [26] and stroke [27].

This research study was designed to test the impact of Prolibra, a dairy-derived ingredient containing whey proteins, peptides and milk minerals, on weight loss, fat loss and lean muscle retention in obese individuals. Our hypothesis was that by purifying the active ingredients from milk (high leucine proteins, peptides and milk minerals) a supplement could be developed that would have a positive impact on fat loss, aid in retaining lean muscle and retain bone mineral content without needing to increase dietary protein intake above the recommended RDI (0.8 g/kg/day). The objective of the trial was to evaluate the effect of a Prolibra beverage on weight loss, body composition and anthropometric measurements over a 12-week period compared to a control beverage.

## Methods

### Subjects

One-hundred and fifty-eight subjects were recruited for this study through local advertising. Subjects were 25–50 years old with a body mass index (BMI) of 30–42 kg/m^2^. Subjects who were pregnant, lactating or at risk for becoming pregnant as well as subjects with digestive disorders, diabetes, hypertension, cardiovascular disease, eating disorders or other illnesses were excluded from the study. Subjects consuming more than one dairy serving per day were counseled to limit dairy intake to one serving per day. The Quorum Institutional Review Board (Seattle, WA) approved the study protocol, informed consent form, subject informational materials and advertisements before subject recruitment. Each subject provided voluntary written consent before initiating any clinical trial related activities.

### Diets

Subjects recorded their food intake (without changing their usual dietary regimen) for five days during two weeks (i.e. Monday, Wednesday, Friday, Tuesday, and Thursday). Subjects returned to the clinic for diet assessment and were randomly assigned to a control group (*n *= 53) or to the Prolibra group (*n *= 53) or a third experimental arm (n = 52, data not shown). This report describes the results from the control group versus the Prolibra group.

Subjects were assigned a diet plan with a certain number of servings for various food groups similar to the standard paradigm set by the American Heart Association [28]. Subjects were counseled to reduce caloric intake by 500 calories per day. Individual diets were assigned based on the subject's Resting Metabolic Rate (RMR) using the formula: RMR × 1.3 – 500. Resting Metabolic Rate was measured by indirect calorimetry using an open-circuit ventilated-hood system. Except for water, subjects fasted for 12 hours prior to the RMR measurements. Subjects rested for 15 minutes prior to RMR measurement and were then placed alone in a recumbent position in a quiet, dimly lit, temperature-controlled room where they underwent 25 minutes of respiratory sampling under the hood. The metabolic monitor recorded energy expenditure readings in one-minute intervals. The final 20 minutes of readings were averaged to arrive at the RMR for that visit. To avoid over estimating energy expenditure, the RMR data were reviewed together with the 2-week baseline diet diaries and subjects were interviewed regarding their physical activity levels before prescribing the diet. All subjects were counseled to keep their physical activity level constant throughout the trial. Subjects were instructed to restrict dairy consumption to ≤ 1 serving per day and total calcium intake to less than 500 mg/day.

The composition of the planned diet was approximately 55% of calories as carbohydrate, 15% as protein, and 30% as fat. These percentages were distributed into 3 meals and 2 snacks per day. The servings were represented in terms of exchanges and a list was provided for the subject outlining appropriate portion sizes and serving suggestions. A sample diet showing the distribution of servings from each macronutrient group was discussed with each subject. A broad range of diet instruction sheets (including 1000, 1200, 1400, 1600, 1800, 2000, 2200, and 2400 calorie diets) were used to direct subjects to comply with their specific diet. Subjects were also given diet diaries to record their food consumption along with reading materials including a grocery foods list, tips for dining out and tips for dieting success. Subjects were instructed that the anticipated weight loss was one pound per week on this diet plan. The composition of the diet combined with the Prolibra supplement produced a carbohydrate to protein ratio of 2.4 in the Prolibra group and a carbohydrate to protein ratio of 3.6 in the control group. Table 1 contains the baseline characteristics for both groups.

**Table 1:** Subject characteristics at baseline (mean ± standard error).

	Control group (n = 28)	Prolibra group (n = 31)	Control group (n = 19)	Prolibra group (n = 23)
	Completers		Responders	
Age (years)	42.0 ± 1.2	43.6 ± 1.1	42.5 ± 1.5	43.6 ± 1.0
Height (cm)	166.4 ± 1.4	166.6 ± 1.3	167.2 ± 1.6	166.7 ± 1.5
Weight (kg)	98.0 ± 2.2	98.9 ± 2.1	100.6 ± 3.0	100.0 ± 2.8
BMI (kg/m2)	35.4 ± 0.7	35.7 ± 0.7	35.9 ± 0.8	35.9 ± 0.8
Waist circumference (cm)	101.1 ± 2.0	105.1 ± 1.9	102.0 ± 1.1	103.8 ± 1.9
Hip circumference (cm)	120.3 ± 1.9	120.1 ± 1.8	122.7 ± 2.2	121.0 ± 2.3

Subjects completed diet diaries on at least five days each month and clinic staff evaluated and discussed the diet dairies at each visit with the subject to assist each subject in controlling their calories, physical activity and calcium intake. Concerns and questions were addressed and eating patterns were discussed.

### Measurements

Subjects were weighed at weeks 0, 4, 8 and 12 in a paper exam gown using a 752 Healthometer Professional-752KL digital scale (Sunbeam, Boca Raton, Fl) and waist and hip circumferences as well as vital signs were measured at weeks 0, 4, 8, and 12. Total body fat, lean muscle tissue and trunk fat were measure by dual energy x-ray absorptiometry (Lunar Prodigy Advance Plus, General Electric, Madison, WI) at weeks 0 and 12. Resting metabolic rate was determined by indirect calorimetry using a TrueOne 2400 Metabolic Measurement system (Parvo Medics Inc., Yorba Sandy, Utah). Total body water was measured using a Quantum II bioelectric impedance analysis instrument (RJL Systems, Clinton Township, MI). Venous blood samples were collected from each subject at weeks 0 and 12 and a chemistry profile, lipid profile, insulin and complete blood counts were obtained (Quest Diagnostics Laboratory, Minneapolis, MN). Waist and hip circumference measurements were also performed with the subjects in an exam gown using a Tech-Med (model #4414) measuring tape according to the following technique: waist circumference was obtained at the midpoint between the level of the lowest rib and the top of the anterior iliac crest and hip circumference was obtained at the level of largest diameter below the anterior iliac crest.

### Supplements

Each subject was instructed to consume one supplement 20 minutes before breakfast and one supplement 20 minutes before dinner. The supplement was in the form of a sachet containing a chocolate flavored ready to mix drink and each subject was provided a hand shaker for mixing the drink. The product was mixed with 8 oz of water in a hand shaker and then consumed. Total protein in the supplements was measured using Kjeldahl (AOAC 945.01) with a conversion factor of 6.38. Total carbohydrate was measured using the phenol-sulfuric acid method [29]. Total ash was measured using AOAC 954.46. Calcium content was measured using AOAC 965.09/975.03AA. Total fat was measured using the Mojonneir method (AOAC 989.05). The nutritional characteristics of the unflavored Prolibra are found in Table 2. The Prolibra supplement contained 10 grams of protein per serving as a combination of intact whey protein and peptides. It also contained minerals that were purified from milk. The control group received an iso-caloric beverage containing maltodextrin. Compliance with the study protocol was assessed by supplement count and diet diary review. Subjects were also contacted by telephone between visits to review diet and supplement compliance and to answer any questions.

**Table 2:** Composition of Prolibra.

Component	Daily Intake
**Protein (g)**	**20**
Leucine (g)	2.24
Isoleucine (g)	1.44
Valine (g)	1.26
**Total BCAA**	**4.94**
Lysine	1.91
Cysteine	0.50
Methionine	0.41
Tryptophan	0.44
Phenylalanine	0.64
Histidine	0.37
Threonine	1.59
Tyrosine	0.62
**Lipid (g)**	**0.12**
**Carbohydrate (g)**	**0.83**
Lactose (g)	0.27
**Minerals**	
Calcium (mg)	482
Phosphorus (mg)	253
Sodium (mg)	213
Potassium (mg)	100
Magnesium (mg)	50
Zinc (ug)	60

### Statistical analysis

The statistical analysis was generated using SAS (Copyright, SAS Institute Inc. Cary, NC, USA.). Baseline differences were determined using one-way ANOVA. Differences between groups at week 12 were determined using one-way analysis of covariance with the baseline data as the covariate (ANCOVA). If a significant group effect was found, post hoc two-group pairwise comparisons based on estimated marginal means were done using the Least Significant Difference (LSD) test. Differences within groups were determined using a two-sided paired t-test. Both a completer analysis and responder analysis was performed. Those that completed the study were included in the completer analysis and subjects that lost at least 2.25 kg of body weight were included in the responder analysis.

## Results

### Subject attrition

Forty-seven (47) subjects withdrew from the study. Among the 22 subjects who withdrew from the treatment group: 2 subjects were withdrawn due to the development of an intercurrent illness/medical condition (one had cellulitis secondary to a bug bite and one persistent pyelonephritis); 1 subject withdrew because she felt the test article had unpleasant properties; 10 subjects withdrew for clinical trial related reasons including that the trial activities took too much time or their personal situation changed at home or at work; 3 subjects were withdrawn for failure to maintain adequate compliance with the clinical trial protocol; 1 subject did not meet the entry criteria; 1 subject was diagnosed with hypothyroidism during the trial and was withdrawn because they used a non-approved medication during the trial (Levothyroxine); 1 subject was withdrawn because she had received the test articles but returned all packages unused; and 3 subjects were lost to follow-up. Among the 25 subjects who withdrew from the placebo group: 12 withdrew for clinical trial related reasons including that the trial activities took too much time or their personal situation changed at home or at work; 9 were withdrawn for failure to maintain adequate compliance with the clinical trial protocol; 1 subject was withdrawn because she no longer met inclusion criteria after a 15 pound weight fluctuation between the first and second visits; 1 subject was withdrawn because she became pregnant during the trial, and 2 subjects were lost to follow-up.

### Dietary analysis

At baseline, the control and Prolibra groups were consuming comparable amounts of carbohydrate, protein, fat and calcium (Table 3). Table 4 shows the macronutrient intake with and without the supplement during the dieting phase of this trial. When the dietary records alone were analyzed we did not find significant differences in macronutrient intake during dieting between the Prolibra and control groups in the completer or responder analysis when the supplement was excluded from the analysis. Including the supplement provided a significant shift in carbohydrate to protein ratio.

**Table 3:** Daily food intake at baseline (mean ± standard error)

	Baseline food intake			
	Completers		Responders	
	Control group	Prolibra group	Control group	Prolibra group

Carbohydrate (g)	211 ± 10	222 ± 11	219 ± 12	219 ± 12
Protein (g)	74 ± 4	73 ± 3	82 ± 4	73 ± 4
Fat (g)	71 ± 5	75 ± 5	74 ± 5	72 ± 6
EtOH (calories)	50 ± 16	38 ± 16	30 ± 9	50 ± 21
Calcium (mg)	381 ± 36	372 ± 30	370 ± 39	359 ± 40
g protein/kg body weight*	0.76	0.74	0.82	0.74
Carbohydrate/Protein ratio	2.9	3.0	2.7	3.0

*Initial body weight was used for this calculation.

**Table 4:** Daily food intake during dieting (mean ± standard error).

	Food intake during dieting*			
	Completers		Responders	
	Control group	Prolibra group	Control group	Prolibra group

Carbohydrate (g)	182 ± 9	178 ± 8	174 ± 13	178 ± 11
Protein (g)	58 ± 2	57 ± 3	56 ± 3	60 ± 5
Fat (g)	47 ± 3	49 ± 3	43 ± 4	47 ± 5
EtOH (calories)	20 ± 7	13 ± 5	11 ± 6	16 ± 6
Calcium (mg)	317 ± 29	275 ± 27	242 ± 26	287 ± 33
g protein/kg weight**	0.61	0.60	0.58	0.63
Carbohydrate/Protein ratio	3.1	3.1	3.1	3.0
**With supplement**				
g protein/kg weight	0.61	0.81	0.58	0.84
Carbohydrate/Protein ratio	3.6	2.4	3.6	2.3

*The nutritional supplements were not included in the dietary calculations.

**Final body weight was used for this calculation.

### Body composition

No baseline differences were found between groups for any body composition parameters (Table 1). After 12 weeks there were significant differences (Table 5). Weight loss was consistently higher in the Prolibra subjects and DEXA analyses showed that the weight loss in the Prolibra group was primarily the result of losing body fat. Prolibra subjects lost significantly more body fat compared to control subjects in both the completer (2.81 kg vs. 1.62 kg *P *= 0.03) and responder (3.63 kg vs. 2.11 kg, *P *= 0.01) analysis. The Prolibra subjects lost significantly less lean muscle mass compared to control subjects in the responder analysis (2.41 kg vs. 1.07 kg, *P *= 0.02); however the completer analysis did not show a significant difference between Prolibra and control subjects (1.55 kg vs. 0.75 kg, *P *= 0.11). Fat loss to lean loss ratio (kg fat loss/kg of lean loss) for Prolibra subjects was higher compared to the control in both the completer (3.75 vs. 1.05) and responder (3.39 vs. 0.88) analysis.

**Table 5:** Weight loss, fat loss and lean loss over 12 weeks (mean ± standard error).

	Completers		Responders	
	Control	Prolibra	Control	Prolibra
Number of subjects	28	31	19	23
Weight loss (kg)	3.24 ± 0.47^#^	3.82 ± 0.55^#^	4.56 ± 0.41^#^	5.20 ± 0.47^#^
% Increase total body water	0.50 ± 0.59^NS^	1.21 ± 0.51^#^	1.00 ± 0.52^NS^	1.40 ± 0.66^#^
Decrease in REE (kCal)	76 ± 52^NS^	40 ± 38^NS^	147 ± 69^#^	37 ± 60^NS^
Centimeters lost (waist)	5.34 ± 0.97^#^	6.22 ± 0.84^#^	6.15 ± 1.20^#^	7.42 ± 0.86^#^
Centimeters lost (hip)	4.90 ± 0.84^#^	4.20 ± 1.55^#^	5.41 ± 1.04^#^	4.47 ± 2.03^#^
Fat mass lost (kg)	1.62 ± 0.33^#*^	2.81 ± 0.38^#*^	2.11 ± 0.44^#*^	3.63 ± 0.40^#*^
Lean mass lost (kg)	1.55 ± 0.39^#^	0.75 ± 0.34^#^	2.41 ± 0.44^#*^	1.07 ± 0.37^#*^

# statistically significant (*P *< 0.05) result within group.

* statistically significant (*P *< 0.05) result between group.

^NS ^Not significant

### Blood chemistry

Table 6 contains the changes in blood profiles during the study for the completer analysis. Similar trends were observed in the responder analysis. There was a significant within group decrease of cholesterol for the treatment group. There was also a significant decrease in blood urea nitrogen in the control group. Tables 5, 6 summarize the significant within group and between group differences.

**Table 6:** Change in blood profiles during dieting based upon completers analysis (mean ± standard error).

**Blood Chemistries**	**Control**	**Prolibra**
Triglycerides (mg/dl)	-7.5 ± 16	-16.9 ± 10
Cholesterol (mg/dl)	-2.21 ± 4	-9.26 ± 4^#^
High Density Lipoproteins (mg/dl)	0.93 ± 1	-1.06 ± 1
Low Density Lipoproteins (mg/dl)	-0.32 ± 4	-5.43 ± 4
Blood Urea Nitrogen (mg/dl)	-1.32 ± 0.5^#*^	0.1 ± 0.6*
Creatine (mg/dl)	0 ± .02	0.01 ± .01
Albumin (g/dl)	0 ± 0.4	0.03 ± .03
Globulin (g/dl)	-0.07 ± .05*	0.05 ± 0.5*
Alkaline Phosphatase (u/l)	-0.96 ± 1.3	3.52 ± 2.6
Aspartate amino transferase (u/l)	-0.64 ± 0.51	-0.13 ± 0.94
Alanine amino transferase (u/l)	-1.64 ± 0.80^#^	-0.94 ± 1.01
Hemoglobin (g/dl)	-0.12 ± 0.18	0.15 ± 0.10

**Immune Parameters **(Thous/mm3)	**Control**	**Treatment**

White Blood Cells	-0.77 ± 0.32	-0.52 ± 0.26
Platelet Count	-16.54 ± 4.34^#^	-13.13 ± 5.72^#^
Absolute Neutrophils	-0.42 ± 1.54	0.08 ± 1.19
Absolute Lymphocytes	0.12 ± 1.32	0.29 ± 0.94
Absolute Monocytes	0.19 ± 0.22	-0.13 ± 0.29
Absolute Eosinophils	0.01 ± 0.17	-0.27 ± 0.27
Absolute Basophils	0.12 ± 0.08	0.03 ± 0.05

# Significant difference (*P *< 0.05) within group (Time 0 versus Time 12 weeks)

* Signficant difference (*P *< 0.05) between group at 12 weeks

## Discussion

Supplementation with Prolibra during dieting increased the loss of body fat and the retention of lean muscle mass compared to supplementation with an isocaloric control that had a lower calcium and lower protein content. Prolibra appears to preserve lean muscle and may partition the weight loss predominantly towards fat at a lower protein dose, 20 grams per day. Being able to target body fat while retaining lean muscle provides a healthy scenario for weight loss and the potential to decrease body fat.

Layman et al. [5] compared diets with differing carbohydrate to protein ratios. Their control group consumed a carbohydrate to protein diet of 3.5:1 with 0.8 grams/kg/day protein intake compared to a treatment group that consumed a 1.5:1 diet with 1.5 grams/kg/day protein. These two groups were calorically restricted to approximately 1,700 cal/day. No significant difference in weight lost was found between the two groups; the high protein group showed a partitioning of the weight loss that preserved lean and targeted fat loss. In our study, with an incremental intake of 20 grams protein per day in the Prolibra group (total intake 0.8 g/kg/day), we observed fat-directed weight loss of 79% (fat loss/(lean loss + fat loss) × 100) compared to 87% reported in the high protein group of the Layman study with an intake of 1.5 g protein/kg/day.

## Conclusion

Subjects in both the control and treatment group lost a significant amount of weight with a 500 calorie reduced diet. Subjects taking Prolibra lost significantly more body fat and showed a greater preservation of lean muscle compared to subjects consuming the control beverage. Because subjects taking Prolibra lost 6.1% of their body fat mass, and because a 5% reduction of body fat mass has been shown to reduce the risk of obesity related disease [3,30], the results have practical significance.

## Competing interests

The study was funded by Glanbia Nutritionals Inc that manufactures and sells Prolibra. Both LSW and EDB are employees of Glanbia Nutritionals Inc.

## Authors' contributions

All authors contributed equally to the design and development of this clinical trial that was conducted at the Minnesota Applied Research Center by JLF and JLZ. MAK was the independent statistician responsible for the statistical analysis. LSW prepared the first draft of the manuscript.
